# Compositional Engineering of *a* La_1-x_Ba_x_CoO_3-δ_-(1-*a*) BaZr_0.9_Y_0.1_O_2.95_ (*a* = 0.6, 0.7, 0.8 and x = 0.5, 0.6, 0.7) Nanocomposite Cathodes for Protonic Ceramic Fuel Cells

**DOI:** 10.3390/ma12203441

**Published:** 2019-10-21

**Authors:** Laura Rioja-Monllor, Carlos Bernuy-Lopez, Marie-Laure Fontaine, Tor Grande, Mari-Ann Einarsrud

**Affiliations:** 1 Department of Materials Science and Engineering, NTNU Norwegian University of Science and Technology, 7491 Trondheim, Norwaytor.grande@ntnu.no (T.G.); 2 SINTEF Industry, 0314 Oslo, Norway; Marie-Laure.fontaine@sintef.no

**Keywords:** proton ceramic fuel cells (PCFC), cathode, exsolution

## Abstract

Compositionally engineered *a* La_1-x_Ba_x_CoO_3-δ_-(1-*a*) BaZr_0.9_Y_0.1_O_2.95_ (*a* = 0.6, 0.7, 0.8 and x = 0.5, 0.6, 0.7) (LBZ) nanocomposite cathodes were prepared by oxidation driven in situ exsolution of a single-phase material deposited on a BaZr_0.9_Y_0.1_O_2.95_ electrolyte. The processing procedure of the cathode was optimized by reducing the number of thermal treatments as the single-phase precursor was deposited directly on the electrolyte. The exsolution and firing of the cathodes occurred in one step. The electrochemical performance of symmetrical cells with the compositionally engineered cathodes was investigated by impedance spectroscopy in controlled atmospheres. The optimized materials processing gave web-like nanostructured cathodes with superior electrochemical performance for all compositions. The area specific resistances obtained were all below 12 Ω·cm^2^ at 400 °C and below 0.59 Ω·cm^2^ at 600 °C in 3% moist synthetic air. The resistances of the nominal 0.6 La_0.5_Ba_0.5_CoO_3-δ_-0.4 BaZr_0.9_Y_0.1_O_2.95_ and 0.8 La_0.5_Ba_0.5_CoO_3-δ_-0.2 BaZr_0.9_Y_0.1_O_2.95_ composite cathodes were among the lowest reported for protonic ceramic fuel cells cathodes in symmetrical cell configuration with ASR equal to 4.04 and 4.84 Ω·cm^2^ at 400 °C, and 0.21 and 0.27 Ω·cm^2^ at 600 °C, respectively.

## 1. Introduction

Protonic ceramic fuel cells (PCFCs) have received considerable attention in the past decades since Iwahara and Takahashi investigated the ionic conduction in perovskite oxide materials in 1971 [1]. One of the main advantages of PCFCs over solid oxide fuel cells (SOFCs) is the lower operating temperature (350–600 °C) because of the lower activation energy for proton conduction compared to oxygen ion conduction [2,3]. Moreover, the formation of the water vapor reaction product at the cathode side in the PCFCs prevents fuel dilution. Yttrium-doped barium zirconates and cerates are among the most studied electrolyte candidates because of their high bulk proton conductivity [4,5]. The anode materials are typically cermets of the selected electrolyte material and nickel as it exhibits excellent catalytic activity and electrical conductivity [6,7]. Upon lowering of the operating temperature of PCFCs, the cathode has been identified as the performance-limiting component and the search of suitable cathodes has been in focus [8,9,10]. The most studied cathode materials for PCFCs are those commonly used in SOFCs such as La_0.8_Sr_0.2_MnO_3_ (LSM) [11,12], La_1-x_Ba_x_CoO_3-δ_ (LBC) [13,14,15,16], La_0.6_Sr_0.4_Co_0.2_Fe_0.8_O_3-δ_ (LSCF) [17,18], and Ba_0.5_Sr_0.5_Co_0.8_Fe_0.2_O_3-δ_ (BSCF) [19]. However, these cathodes without considerable proton conductivity restrict the reaction sites to the electrolyte/cathode interface [20]. In order to extend the active sites where the protons and electrons can interreact, a material with mixed electron and proton conductivity would be preferable [21]. In addition, oxygen vacancies are a prerequisite to induce the proton conductivity by dissolution of water. The triple conducting materials BaCo_0.4_Fe_0.4_Zr_0.1_Y_0.1_O_3-δ_ reported by Duan et al. [9,22] and BaGd_0.8_La_0.2_Co_2_O_6-δ_ reported by Strandbakke et al. [23] with mixed oxygen ion/electron/proton conductivity have also been shown as suitable cathode materials for PCFCs. Still, infiltration techniques [9] or functional layers [23] were necessary in order to improve the performance of these cathodes. Deposition by infiltration into a porous backbone enables the preparation of finely nano-structured composites, but the process is time consuming and to prevent the formation of secondary phases can be challenging [18]. Further studies on these materials are progressing to determine their long-term stability and further optimize these compositions and microstructure, in particular, to reduce their large thermal expansion [18]. Alternatively, we have recently developed a novel synthesis method to prepare La_0.5_Ba_0.5_CoO_3-δ_-BaZrO_3_ (LB-BZ)-based composite cathodes using in situ exsolution of a single phase oxide material under oxidizing conditions [24,25,26]. The exsolution of nanoparticles at reducing conditions has already been used by several groups to design new anodes [27,28,29,30,31]. However, as the cathode of PCFC operates under oxidizing conditions, our alternative approach enables the formation of suitable materials at the operating conditions of a cathode. In our previous work, La_0.5_Ba_0.5_CoO_3-δ_ material was tested as a single phase cathode for PCFC and exhibited excellent performance of 7.4 and 0.16 Ω·cm^2^ at 400 and 600 °C in 3% moist synthetic air but there was no indication of proton conduction in this material [16]. La_1-x_Ba_x_CoO_3-δ_ materials, with excellent oxygen ion and electronic conductivity [13,32,33,34], and Y-doped barium zirconate materials (BZY), with fast proton conduction, are state-of-the art cathode and electrolyte in PCFCs [5,35,36]. As required for electrolytes, BZY materials show low electronic conductivity which is not beneficial for the use as component in a composite cathode. The exsolution synthesis approach based on in situ driven decomposition and the possibility to induce phase decomposition by red-ox chemistry enables the preparation of Co doped BZ phase [24,25] that exceeds the electronic percolation threshold. In addition, the solid solubility of Co in the BZ-phase increases the electronic conductivity of this phase [24,25,26]. In our previous work, the amount of the catalytic active LB-phase for the oxygen reduction reaction (ORR) was 40 mol%, which was lower than the nominal composition. The percolation of the two constituents is important as the best performing cathodes are usually mixtures of 50:50 or 60:40 O^2−^/e^−^ conductor and H^+^ conductor material, respectively [37,38]. Finally, proton conduction in perovskite materials seems to be enhanced when the A-site is occupied by the strongly basic Ba which enhances the hydration of oxygen vacancies in the crystal structure [5,39].

In this work, compositional engineering of PCFC cathodes was performed focusing on the composite phase ratio between the LB and BZ phases as well as the Ba content of the LB phase. The influence of these parameters on the electrochemical performance of the cathodes was investigated by increasing the nominal LB phase ratio from 60 up to 80 mol%. In addition, the Ba content in the La_1-x_Ba_x_CoO_3-δ_ phase was varied from the nominal x = 0.5 to 0.6 and 0.7. The new cathode materials were characterized by X-ray diffraction and the microstructure of the cathodes was investigated by scanning electron microscopy. The electrochemical performance was analyzed in symmetrical cell configuration by electrochemical impedance spectroscopy. The results are analyzed and discussed with correlation to the composition and microstructure of the cathodes.

## 2. Materials and Methods

Composite cathodes with nominal compositions 0.6 La_0.5_Ba_0.5_CoO_3-δ_-0.4 BaZr_0.9_Y_0.1_O_2.95_, 0.7 La_1-x_Ba_x_CoO_3-δ_-0.3 BaZr_0.9_Y_0.1_O_2.95_ (x = 0.5, 0.6, 0.7), and 0.8 La_1-x_Ba_x_CoO_3-δ_-0.2 BaZr_0.9_Y_0.1_O_2.95_ (x = 0.5, 0.6) were synthesized by a modified Pechini method followed by in situ oxidation driven exsolution decomposition of a single phase material. Table 1 summarizes the compositions of the prepared materials as well as the nomenclature used. The composites consist of a La_1-x_Ba_x_CoO_3-δ_-based phase (marked as LB) and BaZr_0.9_Y_0.1_O_2.95_-based phase (marked as BZ). The nomenclature contains two numbers, the first one represents the nominal mole fraction of the LB phase and the second one represents the nominal atomic fraction of Ba in the LB phase.

**Figure 1:**
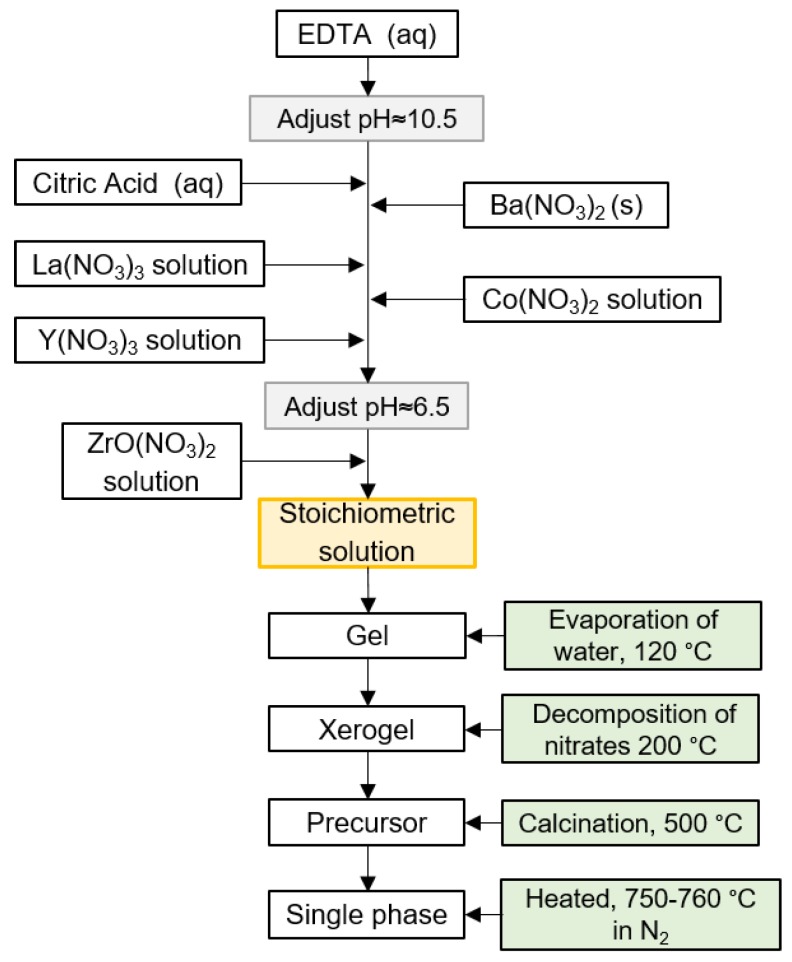
Flow chart of the modified Pechini synthesis of La_1-x_Ba_x_CoO_3-δ_-BaZr_1-z-y_Y_z_Co_y_O_3-δ_-based composites. The precursor is annealed in N_2_ to form a single-phase material stable at room temperature.

The cation precursors for the synthesis of the composites were barium nitrate (Ba(NO_3_)_2_, >99.999%), lanthanum nitrate hexahydrate (La(NO_3_)_3_·6H_2_O, >99.99%), zirconyl nitrate hydrate (ZrO(NO_3_)_2_·xH_2_O, >99%), cobalt nitrate hydrate (Co(NO_3_)_2_·6H_2_O, >99.999%), and yttrium nitrate tetrahydrate (Y(NO_3_)_3_·4H_2_O, >99.8%). Ethylenediaminetetraacetic acid (EDTA, >99%) as well as citric acid (CA, >99%) were used as complexing agents. All the starting materials were bought from Sigma-Aldrich. The materials were prepared as shown schematically by the flow chart in Figure 1, based on our previous work [24,25]. Stoichiometric amounts of cation precursors were mixed to give the nominal compositions given in Table 1. The sols were gelled on a hot plate at 120 °C, decomposed at 200 °C and calcined at 500 °C (except for LBZ65, which was calcined at 450 °C) in order to prepare the organic-free oxide precursor. The precursor powders were uniaxially pressed into pellets at 50 MPa and annealed in N_2_ atmosphere for 8 h at 715 °C for LBZ65, at 750 °C for LBZ75, LBZ76 and LBZ77, and at 760 °C for LBZ85 and LBZ86, in order to achieve a single-phase material. The annealed pellets of the single-phase materials were ground in a mortar in order to obtain a fine powder. All the thermal treatments were performed using 2 °C/min cooling and heating rates. A fraction of the single phases was uniaxially pressed into pellets at 50 MPa and annealed for 2 h at 1100 °C in ambient air for structural characterization (marked as ex situ exsolved).

**Figure 2:**
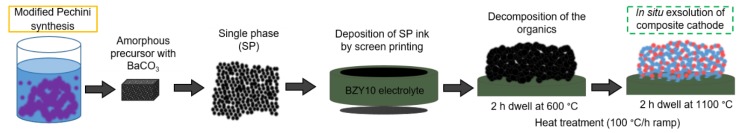
Schematic illustration of the in situ exsolution of La_1-x_Ba_x_CoO_3-__δ_-BaZr_1-z-y_Y_z_Co_y_O_3-δ_ cathode materials on the surface of the electrolyte. The cathode processing and exsolution of the composite occur in one step.

Electrolyte supported symmetric cells were produced by screen printing. The inks were prepared by mixing: single phase powder (3 g), dispersant (1 g, 20 wt.% of Solsperse Lubrizol 28,000 in terpineol), and binder (0.2 g, 5 wt.% Heraeus V-006 in terpineol) in a mortar and ground until homogenization [9]. The BaZr_0.9_Y_0.1_O_2.95_ (BZY10) electrolyte was prepared as described by Sazinas et al. [40,41]. Green cylindrical pellets (12 mm^ø^) were made and sintered in a sacrificial powder bed (BaZr_0.8_Y_0.2_O_2.9_ with 10 wt.% BaCO_3_) at 1600 °C for 10 h in ambient air with 10 °C/min heating rate. The surfaces of the sintered electrolyte pellets were ground with SiC papers to reach a final thickness of 1 mm. The inks were screen printed on both sides of the electrolyte and fired for 2 h at 1100 °C in order to exsolve the single-phase materials into the composite cathode materials (marked as in situ exsolved), with 2 h dwell at 600 °C to assure the removal of the organics prior to exsolution. Gold paste was applied onto the cathodes and platinum was employed as a conducting wire. Figure 2 illustrates the synthesis and processing of the composite cathodes via the in situ exsolution of the single-phase material deposited onto the electrolyte.

The prepared powders and the cathodes in symmetric cell configuration before and after electrochemical impedance spectroscopy (EIS) testing were analyzed by X-ray diffraction (XRD) using a Bruker D8 DaVinci (Billerica, MA, USA) equipped with Lynxeye^TM^ detector and Cu*K*α radiation. Unit cell parameters were refined by the Rietveld method using Bruker AXS TOPAS software v5 (Billerica, MA, USA). The lattice parameters of the single-phase materials annealed in N_2_ were obtained by profile fitting of a Pm-3m cubic perovskite structure. The XRD patterns of ex situ exsolved cathodes were refined using two cubic perovskite phases with Pm-3m space group, La_1-x_Ba_x_CoO_3-δ_ and BaZr_1-z-y_Y_z_Co_y_O_3-δ_. The structural data of the nominal compositions was used as the starting point in the Rietveld refinements. Lattice parameters and the x, y, and z variables were refined.

The microstructure and adhesion of the composite cathodes were studied by field emission scanning electron microscopy (SEM, Zeiss ultra 55, Jena, Germany). Cross sections of non-polished symmetrical cells before and after EIS testing were examined. The adhesion of the cathodes to the electrolyte was evaluated by visual investigation and verified by SEM. To further qualify the adhesion of the cathodes to the electrolyte, a “carbon tape” test was performed where the conductive carbon tape used to mount samples for SEM analysis was used in an attempt to peel off the cathode layers. Dispersions of ground LBZ65 and LBZ85 composite materials in ethanol were dropped on holey carbon coated copper grids for transmission electron microscopy (TEM). TEM images were recorded on a double Cs corrected coldFEG Jeol JEM ARM200F (JEOL Ltd., Tokyo, Japan), operated at 200 kV and equipped with a 100 mm^2^ Centurio SDD (0.98 sr solid angle) for X-ray energy dispersive spectroscopy (EDX) and a Quantum GIF for dual electron energy loss spectroscopy (EELS). The phases of the composite were identified by measuring the d_hkl_ distances. An area of interest was extracted from the high-resolution images (HR-TEM) with fast Fourier transform analysis, and the average distance was calculated over more than ten consecutive *hkl* planes, using DigitalMicrograph with Gatan Microscopy Suite software (GMS 3, DigitalMicrograph®, Pleasanton, CA, USA).

Electrochemical impedance spectroscopy (EIS) of symmetrical cells was measured in moist (*p*H_2_O = 0.03 atm) synthetic air from 400 to 600 °C, in temperature intervals of 50 °C (with a cooling rate of 1 °C/min and 8 h dwell before measurement) using a ProboStat^TM^ (NorECs AS, Oslo, Norway) set-up and an Alpha A (Novocontrol Technologies) impedance analyzer. The signal amplitude was 50 mV under open circuit voltage (OCV) in the 10^−2^–10^6^ Hz frequency range. Synthetic air was connected to a bubbler containing distilled water at 25 °C in order to achieve 3% moist atmosphere. The experimental data was fitted using Zview software with the equivalent circuits LR_s_(R_1_Q_1_)(R_2_Q_2_) or LR_s_(R_1_Q_1_)(R_2_Q_2_)(R_3_Q_3_) with fitting errors below 0.01 Ω·cm^2^. R*_i_* and Q*_i_* are, respectively the polarization resistance and the constant phase element for the different processes. The ohmic resistance of the bulk electrolyte is represented by R_s_ and the inductance by L. The different processes were attributed to the electrolyte or electrode using the pseudo capacitances (C) [42].

## 3. Results and Discussion

**Figure 3:**
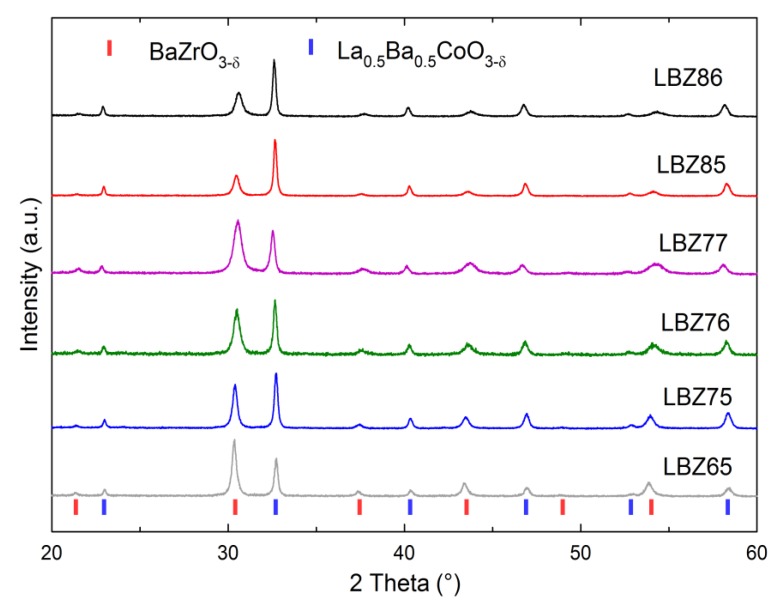
X-ray diffraction (XRD) patterns at room temperature of the composites with nominal composition La_1-x_Ba_x_CoO_3-δ_-BaZr_1-z-y_Y_z_Co_y_O_3-δ_ ex situ exsolved at 1100 °C. Bragg reflections of BaZrO_3-δ_ (red) and La_0.5_ Ba_0.5_CoO_3-δ_ (blue) are shown for comparison.

The XRD patterns of the ex situ exsolved cathodes shown in Figure 3 demonstrate the presence of only two phases for all compositions after calcining the single-phase material at 1100 °C for 2 h in air. A clear difference in the width and intensity of the diffraction lines of the two phases is observed, but only small variations in the position of the Bragg reflections can be noted when compared with the pattern materials, inferring small variations in the lattice cell parameters. In addition, no extra peaks could be detected which confirms the purity of the materials.

**Figure 4:**
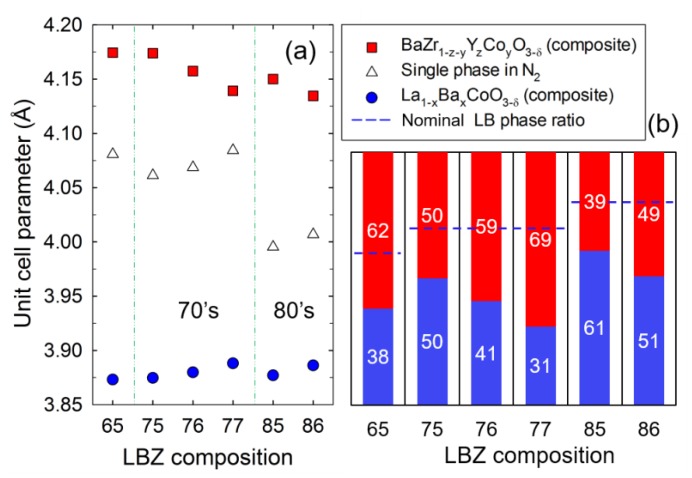
(**a**) Cubic unit cell parameters at room temperature of the single-phase material (open symbols) and the composite phases (red and blue symbols) and (**b**) phase ratio in LBZ materials obtained by Rietveld refinement. Blue dashed lines indicate the LB/BZ nominal phase ratio.

The lattice parameter of the single-phase materials (nominal composition given in Table 1) are included in Figure 4a as well as the unit cell parameters of the two phases of the ex situ exsolved composites. The cell parameters of the single phases decrease with increasing LB content in the composite (LBZ65 > LBZ75 > LBZ85). Increasing the nominal LB phase content leads to a larger content of La and Co^2+^/Co^3+^ (0.745/0.61 Å) relative to Ba, Zr (0.72 Å) and Y (0.9 Å) [43] as seen from the nominal single phase composition given in Table 1. The smaller ionic radii of La^3+^ (1.36 Å) relative to Ba^2+^ (1.61 Å) at the A-site explains the decrease in the cell parameter of LB phase within each series with constant nominal phase composition. On the other hand, the increase in cell parameter within the 70s and 80s families is explained by the larger Ba content in the LB phase (LBZ75 < LBZ76 < LBZ77 and LBZ85 < LBZ86) leading to a higher mole fraction of Ba in the overall single phase material. At the same time, these trends confirm the successful compositional engineering of the single phase and the flexibility of the cubic perovskite to accommodate large compositional variations.

The refined site occupancies of La and Ba at the A-site in the LB phase and Co, Zr and Y at the B-site in the BZ phase are given in Table 2. All the materials consist of a lanthanum-rich LB phase and a cobalt-containing BZ phase as observed in our previous work [24,25]. The cell parameter of the BZ phase decreases with increasing LB phase content (65_BZ_ > 75_BZ_ >> 85_BZ_) as the Co content (Co^3+^ at oxidizing conditions) increases giving a smaller cell parameter. The LB phase has no significant variation in lattice parameter as the refined La and Ba content at the A-site is similar for these three materials (Table 2). For the BZ phase within the 70s and 80s families, a decrease in the cell parameter because of a larger amount of Co occupying the B-site is observed. The cobalt content in the BZ phase found by the Rietveld refinement was y = 0.27 in LBZ75, 0.45 in LBZ77, 0.38 in LBZ85, and 0.51 in LBZ86. On the contrary, the increase in cell parameter observed in LB within the 70s and 80s families can be explained by the slightly larger Ba content in this phase from x = 0.37 to 0.40 in LBZ75-77 and from x = 0.37 to 0.39 in LBZ85-86. These trends are directly affected by the composite phase composition. In our previous studies, we concluded that the mechanism for the exsolution of the single phase is the diffusion of La and Co from the single phase forming the LB phase and hence, the change in the phase composition of the composite [24]. The solid solubility of Co in BZ gives a larger mole fraction of BZ and a La-rich LB phase with respect to the nominal phase composition. The refined phase compositions are given in Figure 4b and the nominal ratios are represented as dashed blue lines.

**Figure 5:**
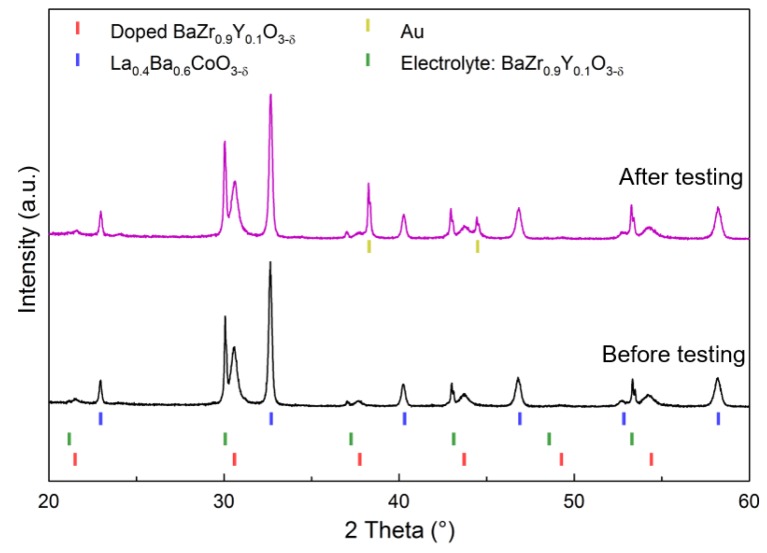
XRD patterns of LBZ86 composite with 0.8La_0.4_Ba_0.6_CoO_3-δ_-0.2BaZr_0.9-_Y_0.1_O_2.95_ nominal composition. The black XRD pattern represents the composite cathode exsolved at 1100 °C on the electrolyte and the purple XRD pattern represents the cathode after EIS characterization in 3% moist synthetic air. Reflections from the gold current collector can be seen after the testing.

The XRD pattern of the in situ exsolved LBZ86 cathode onto the electrolyte (representative for all compositions) is shown in Figure 5. The XRD patterns of cathodes in situ exsolved on the symmetrical cells match well with the ex situ exsolved composites and therefore, the refined composition of the composites is assumed to be the same for both types of cathodes. No changes are observed in the XRD patterns of the cathodes before and after electrochemical characterization as shown in Figure 5.

**Figure 6:**
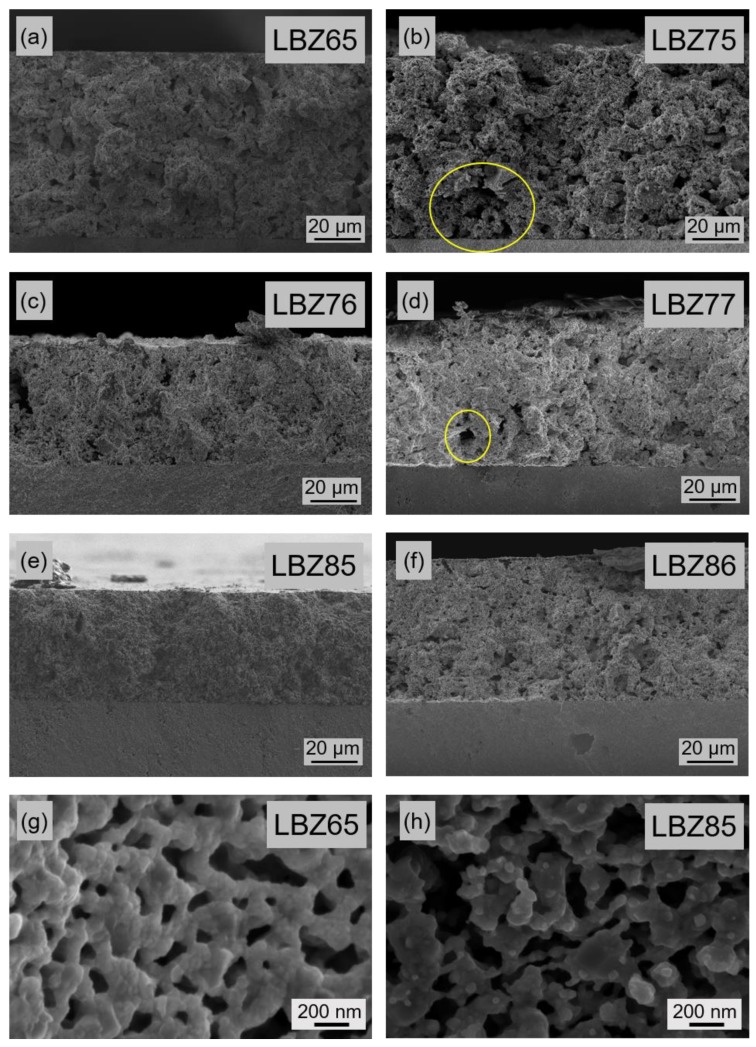
(**a**) to (**f**) Scanning electron microscopy (SEM) micrographs of tested LBZ cathodes in symmetrical cell configuration prepared at 1100 °C. The cells were tested by electrochemical impedance spectroscopy (EIS) from 400 to 600 °C in 3% moist synthetic air. Inhomogeneities in the cathodes are highlighted in yellow. High magnification micrographs of web-like microstructure of LBZ65 (**g**) and LBZ85 (**h**) cathodes after electrochemical testing.

The microstructure of the cross section of the symmetrical cells after electrochemical characterization are presented in Figure 6. The microstructure of the cathodes was similar before and after the electrochemical performance analysis, hence only images from after the testing are presented. Robust, well-adhered and porous cathodes with homogeneous thicknesses in the range from 30 to 45 µm were obtained by screen printing. Homogeneous microstructures were obtained for all the cathodes; however, some large agglomerates or inhomogeneous porosity were observed for LBZ75 and LBZ77 (large voids highlighted in yellow in Figure 6b,d). The cathode microstructure was directly affected by the grinding of the powders and the homogeneity of the ink. Higher magnification of the LBZ65 and LBZ85 cathode microstructures given in Figure 6g,h show agglomerates of 200 nm connected by single grains, grain sizes below 50 nm, and high porosity. These porous cathodes will promote gas diffusion and thus, increase the number of active sites leading to a high number of triple phase boundaries. This web-like microstructure achieved by the in situ exsolution method is representative for all cathode materials [25]. No delamination nor cracks were observed by eye examination of the symmetrical cells after testing and all the samples successfully passed the “carbon tape” test (no cathode residue was observed on the tape).

**Figure 7:**
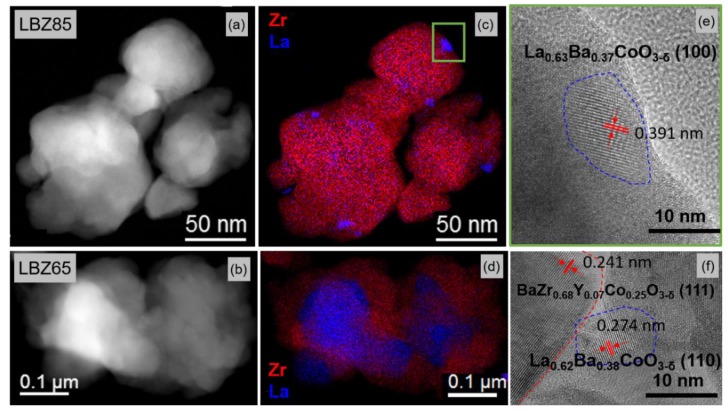
High angle annular dark field scanning transmission electron microscopy (HAADF-STEM) micrographs of (**a**) LBZ85 and (**b**) LBZ65 cathodes. (**c**) EELS combined map of Zr M4,5 edges and La M4,5 edges. (**d**) Energy Dispersive X-Ray Spectroscopy (EDS) combined map of Zr Kα + Lα and La Lα. High resolution TEM magnified region of (**e**) LBZ85 and (**f**) LBZ65. Approximate circumference of the grains are marked as guide for the eye.

A high contiguity between the LB and BZ phases for LBZ65 and LBZ85 materials is observed in the HR-TEM and high angle annular dark field scanning transmission electron microscopy (HAADF STEM) images in Figure 7. In both materials, agglomerates of about 100–250 nm are present containing LB and BZ grains. Figure 7b shows the EELS Zr and La combined map of LBZ85 where the nanoparticles observed by SEM are confirmed to be the LB phase. LBZ85 therefore shows bimodal grain size distribution of the LB phase with larger grains of about 50 nm in addition to the 10-nm nanoparticles decorating the surface of the cathode. Combined Zr and La element EDX mapping of the LBZ65 composite is shown in Figure 7e. LBZ65 consists of a mixture of LB and BZ grains of about 50 nm for both phases. The presence of LB nanoparticles in LBZ85 can be explained by the higher LB phase content (0.61 mole fraction), compared to LBZ65 (0.38 mole fraction). The web-like microstructure observed in SEM was not observed by TEM as the grinding for the sample preparation broke down this microstructure.

**Figure 8:**
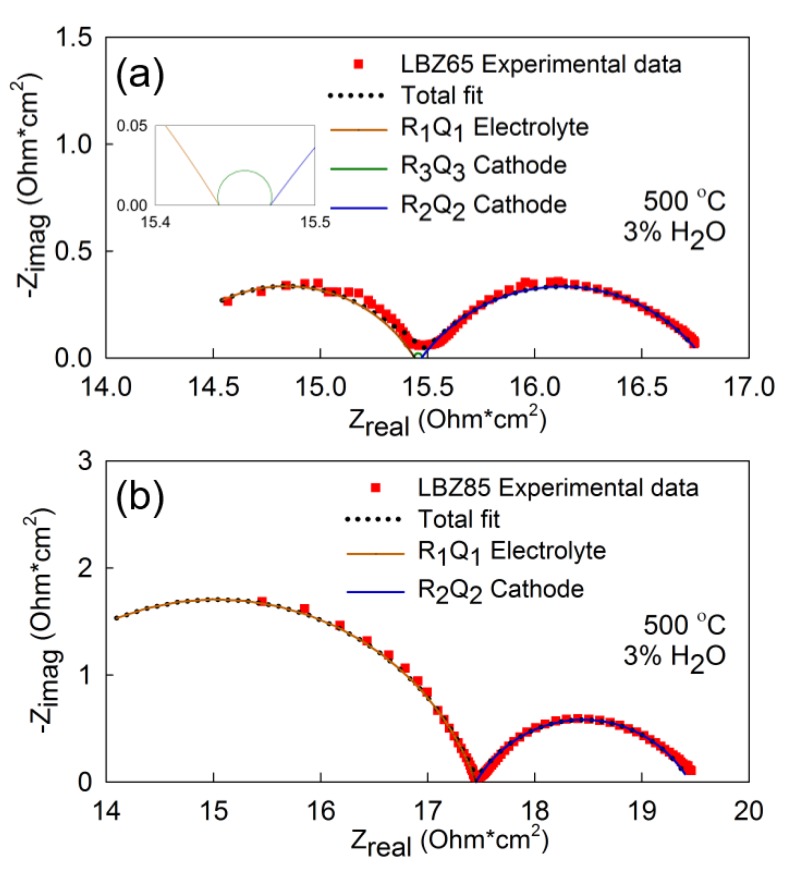
Nyquist plot of (**a**) LBZ65 and (**b**) LBZ85 symmetrical cells with screen printed composite cathodes in synthetic air with p(H_2_O) = 0.03 atm at 500 °C. The inset in (a) shows the magnification of R3Q3 fitting. The symbols correspond to the experimental data and the solid and dotted lines to the fits. The equivalent circuits used for fitting are (a) LRs(R_1_Q_1_)(R_3_Q_3_)(R_2_Q_2_) and (b) LRs(R_1_Q_1_)(R_2_Q_2_).

**Figure 9:**
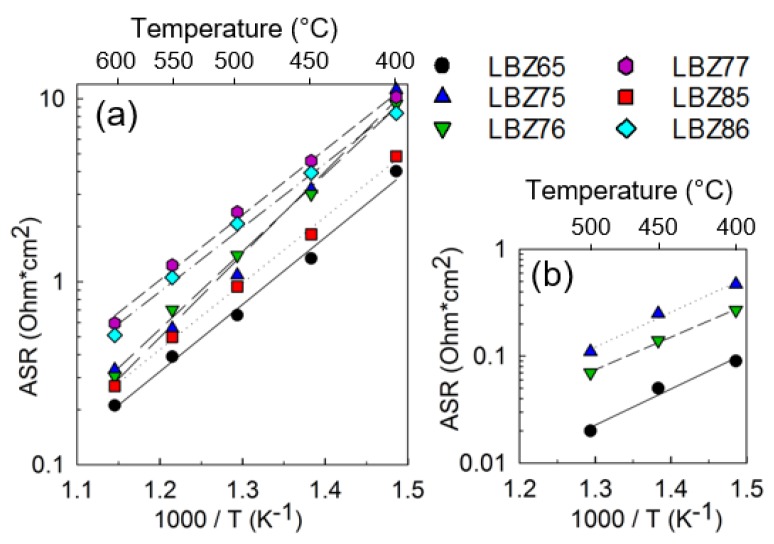
(**a**) Total area specific resistance (ASR) and (**b**) ASR of the (R_3_Q_3_) charge transfer process of the LBZ composite cathodes in symmetric cell configuration in 3% moist synthetic air.

Nyquist plots of symmetrical cells with LBZ65 and LBZ85 cathode materials tested in 3% moist synthetic air at 500 °C and the corresponding fitted models are shown in Figure 8. The deconvolution of the electrochemical response of the symmetrical cells was done using the LR_s_(R_1_Q_1_)(R_3_Q_3_)(R_2_Q_2_) model for LBZ65 (Figure 8a) and the LR_s_(R_1_Q_1_)(R_2_Q_2_) model for LBZ85 (Figure 8b). The LR_s_(R_1_Q_1_)(R_2_Q_2_) model has been utilized to deconvolute the response of all the symmetrical cells except for LBZ65, LBZ75, and LBZ76 at 450 and 500 °C, which were fitted with the LR_s_(R_1_Q_1_)(R_3_Q_3_)(R_2_Q_2_) model. The (R_2_Q_2_) and (R_3_Q_3_) processes were assigned to the electrode with C_2_ ~10^−2^ Fcm^2^ and C_3_ ~10^−4^ Fcm^2^, respectively, while R_1_ was assigned to the electrolyte grain boundary response (C_1_ ~3 × 10^−9^ Fcm^2^) [44]. The (R_2_Q_2_) low frequency response with a high pseudocapacitance is associated to the oxygen adsorption/dissociation steps at the surface and the surface diffusion of the adsorbed oxygen [17,45,46]. (R_3_Q_3_) middle-range frequency response is related to the ionic charge transfer at the electrode/electrolyte interface [47]. The total area specific resistances, ASR, of the cathode composites in symmetric cell configuration in 3% moist synthetic air are shown in Figure 9a. ASRs are calculated as the sum of the resistances attributed to the electrode. All cathode materials showed excellent performance with a total ASR below 12 Ω·cm^2^ and e.g., as low as 4.04 Ω·cm^2^ at 400 °C for LBZ65. LBZ65 and LBZ85 show the best performance with an ASR below 5 Ω·cm^2^ at 400 °C and below 0.3 Ω·cm^2^ at 600 °C. The ASR values with the corresponding total activation energies are given in Table 3. All the cathodes showed total activation energies below 0.9 eV with the lowest value for LBZ86 of 0.70 eV. Cells with LBZ65 and LBZ85 materials showed the best performance and also exhibit low activation energies, 0.73 and 0.71 eV, respectively.

The cathode surface corrected ASR values for the (R_2_Q_2_) and (R_3_Q_3_) processes for the symmetrical cells are included in Table 3 and the ASRs of the charge transfer (R_3_Q_3_) processes of symmetrical cells with LBZ65, LBZ76, and LBZ77 are presented in Figure 9b. The corresponding activation energies varying from 0.64 to 0.60 eV (Table 3) are in accordance with the typical proton migration activation energies (0.6 eV) [45,48]. The pseudo-capacitance of this process (10^−4^ Fcm^2^) may suggest that the rate-limiting step could be associated to the hydrogen dissociation and diffusion [49,50]. The disappearance of the charge transfer contribution for symmetrical cells made with LBZ77, LBZ85, and LBZ86 cathodes indicates that the increase of Ba content in the LB phase as well as the increase of the amount of LB phase in the composite is beneficial for the hydrogen dissociation and diffusion. Experiments at different *p*O_2_ and *p*H_2_O would help to investigate the compositional influence on the rate-limiting step of the cathode electrochemical response.

The oxygen adsorption/dissociation process (R_2_Q_2_) is directly related to the cathode microstructure and the volume fraction of the oxygen active phase as Adler et al. [51] concluded in their study of porous mixed-conducting oxygen electrodes based on oxygen ion conducting electrolytes, using a continuum modeling to analyze the oxygen reduction reaction. Another indication of the microstructural effect on the cathode performance is the pre-exponential factor which was calculated from the Arrhenius plots as described elsewhere [23]. The pre-exponential factor for each cathode material is included in Table 3. A low pre-exponential factor indicates a larger reaction area of the cathode, which can be explained as an increase in the number of catalytic sites [11]. Symmetrical cells with LBZ65, LBZ77, and LBZ86 cathode composites show pre-exponential factors below 1. A larger pre-exponential factor is obtained for LBZ75 (6.56), which is in good agreement with the SEM analysis where large voids were found along the cathode layer. LBZ85 also shows a relatively higher pre-exponential factor (5.40), indicating the possibility to further improve the cathode microstructure and therefore, increase the electrochemical performance of the LBZ85 composite cathode.

The compositional changes of the LBZ materials do not show evident impact on the electrochemical performance as all the cathodes showed excellent performance, similar to the-state-of-the-art materials [9,23]. Special attention should be given to the microstructure in order to extract the compositional contribution to the overall performance. In addition, the possibility to optimize the microstructure of the LBZ85 cathode (pre-exponential factor = 5.40) could lead to further improvements in performance. LBZ65 and LBZ85 cathode materials performed better than the best materials reported in the literature, BaCo_0.4_Fe_0.4_Zr_0.1_Y_0.1_O_3−δ_ (~10 Ω·cm^2^) by Duan et al. [9], La_0.5_Ba_0.5_CoO_3-δ_ (7.4 Ω·cm^2^) in our previous work [16], BaGd_0.8_La_0.2_Co_2_O_6−δ_ (~6 Ω·cm^2^) by Strandbakke et al. [23] at 400 °C at the same experimental conditions. Table 4 shows a comparison of the best materials reported in the literature at 600 °C in 3% moist synthetic air.

## 4. Conclusions

Cathode composites with nominal composition *a* La_1-x_Ba_x_CoO_3-δ_-(1-*a*) BaZr_0.9_Y_0.1_O_2.95_ (LB-BZ) for PCFCs were successfully synthesized and characterized by means of XRD, SEM, and TEM. The increase of LB phase content was achieved from a = 0.38 mole fraction in LBZ65 to a = 0.61 mole fraction for LBZ85 confirmed by XRD Rietveld refinements. The synthesis method developed combined with the one-step exsolution and deposition method allowed to produce homogeneous and porous cathodes with a single grain connected microstructure. Excellent electrochemical performance with an area-specific resistance below 12 Ω·cm^2^ at 400 °C and below 0.59 Ω·cm^2^ at 600 °C was measured for all cathode composites, comparable to the best cathodes reported in the literature. The electrochemical response is dominated by the diffusion or surface related processes at low frequencies. Excellent performing LBZ65 and LBZ85 cathodes with ASR equal to 4.04 and 4.84 Ω·cm^2^ at 400 °C, and 0.21 and 0.27 Ω·cm^2^ at 600 °C are the best reported cathodes in a symmetrical cell configuration. These results demonstrate that LBZ65 and LBZ85 are promising cathodes for PCFCs.

## Figures and Tables

**Table 1:** Nominal composition, nomenclature and annealing temperature in N_2_ of the single-phase material of the LBZ (*a* La_1-x_Ba_x_CoO_3-δ_-(1-*a*) BaZr_0.9_Y_0.1_O_2.95_ (*a* = 0.6, 0.7, 0.8 and x = 0.5, 0.6, 0.7)) compositions.

Code	Nominal Composite Composition	Nominal Single-Phase Composition	Annealing Temperature in N_2_ (°C)
LBZ65	0.6 La_0.5_Ba_0.5_CoO_3-__δ_-0.4 BaZr_0.9_Y_0.1_O_2.95_	La_0.3_Ba_0.7_Co_0.6_Zr_0.36_Y_0.04_O_3-__δ_	715
LBZ75	0.7 La_0.5_Ba_0.5_CoO_3-__δ_-0.3 BaZr_0.9_Y_0.1_O_2.95_	La_0.35_Ba_0.65_Co_0.7_Zr_0.27_Y_0.03_O_3-__δ_	750
LBZ76	0.7 La_0.4_Ba_0.6_CoO_3-__δ_-0.3 BaZr_0.9_Y_0.1_O_2.95_	La_0.28_Ba_0.72_Co_0.7_Zr_0.27_Y_0.03_O_3-__δ_	750
LBZ77	0.7 La_0.3_Ba_0.7_CoO_3-__δ_-0.3 BaZr_0.9_Y_0.1_O_2.95_	La_0.21_Ba_0.79_Co_0.7_Zr_0.27_Y_0.03_O_3-__δ_	750
LBZ85	0.8 La_0.5_Ba_0.5_CoO_3-__δ_-0.2 BaZr_0.9_Y_0.1_O_2.95_	La_0.4_Ba_0.6_Co_0.8_Zr_0.18_Y_0.02_O_3-__δ_	760
LBZ86	0.8 La_0.4_Ba_0.6_CoO_3-__δ_-0.2 BaZr_0.9_Y_0.1_O_2.95_	La_0.32_Ba_0.68_Co_0.8_Zr_0.18_Y_0.02_O_3-__δ_	760

**Table 2:** Nominal (N) and refined (R) composite ratio, A-site occupancy in LB-phase and B-site occupancy of BZ-phase of LBZ composite cathodes. The maximum uncertainty is ±4%.

		La_1-x_Ba_x_CoO_3-δ_ Phase			BaZr_1-z-y_Y_z_Co_y_O_3-δ_ Phase			
		LB Phase Mole	A-Site Occupancy		BZ Phase Mole	B-Site Occupancy		
Code		Fraction	La	Ba	Fraction	Zr	Y	Co
LBZ65	N	0.60	0.5	0.5	0.40	0.9	0.1	0
	R	0.38	0.62	0.38	0.62	0.68	0.07	0.25
LBZ75	N	0.70	0.5	0.5	0.30	0.9	0.1	0
	R	0.50	0.63	0.37	0.50	0.66	0.07	0.27
LBZ76	N	0.70	0.4	0.6	0.30	0.9	0.1	0
	R	0.41	0.62	0.38	0.59	0.51	0.07	0.42
LBZ77	N	0.70	0.3	0.7	0.30	0.9	0.1	0
	R	0.31	0.60	0.40	0.69	0.47	0.08	0.45
LBZ85	N	0.80	0.5	0.5	0.20	0.9	0.1	0
	R	0.61	0.63	0.37	0.39	0.56	0.06	0.38
LBZ86	N	0.80	0.4	0.6	0.20	0.9	0.1	0
	R	0.51	0.61	0.39	0.49	0.45	0.04	0.51

**Table 3:** Cathode area specific resistance (ASR) in Ω·cm^2^ from 400 to 600 °C at pH_2_O = 0.03 atm, total and per process activation energies and pre-exponential factor for the different LBZ cathodes.

	LBZ65		LBZ75		LBZ76		LBZ77	LBZ85	LBZ86
T (°C)	(R_3_Q_3_)	(R_2_Q_2_)	(R_3_Q_3_)	(R_2_Q_2_)	(R_3_Q_3_)	(R_2_Q_2_)	(R_2_Q_2_)	(R_2_Q_2_)	(R_2_Q_2_)
600		0.21		0.33		0.30	0.59	0.27	0.51
550		0.39		0.52		0.70	1.23	0.50	1.06
500	0.02	0.64	0.11	0.98	0.07	1.39	2.40	0.94	2.08
450	0.05	1.28	0.25	2.95	0.14	2.88	4.57	1.82	3.93
400	0.09	3.95	0.47	10.66	0.27	9.08	10.21	4.84	8.34
Process Ea (eV)	0.64	0.72	0.61	0.89	0.60	0.83	0.71	0.71	0.70
Total Ea (eV)	0.73		0.90		0.84		0.71	0.71	0.70
Pre-exponential factor	0.81		6.56		3.03		0.19	5.40	0.19

**Table 4:** Comparison of the cathode area specific resistance from the literature in 3% moist synthetic air at 600 °C in symmetric cell configuration.

Cathode Material	Electrolyte	ASR (Ohm·cm^2^)	Activation Energy (eV)	Ref.
La_0.8_Ba_0.2_CoO_3-__δ_- BaZr_0.6_Co_0.4_O_3-__δ_ (40:60 mol%)	BaZr_0.9_Y_0.1_O_2.95_	0.34	0.92	24
La_0.8_Ba_0.2_CoO_3-δ_- BaZr_0.6_Co_0.4_O_3-δ_ (40:60 mol%) In situ exsolved	BaZr_0.9_Y_0.1_O_2.95_	0.30	0.78	24
LBZ65: La_0.62_Ba_0.38_CoO_3-__δ_- BaZr_0.68_Y_0.07_Co_0.25_O_3-δ_ (38:62 mol%) in situ exsolved	BaZr_0.9_Y_0.1_O_2.95_	0.21	0.73	This work
LBZ85: La_0.63_Ba_0.37_CoO_3-__δ_- BaZr_0.56_Y_0.06_Co_0.38_O_3-δ_ (61:39 mol%) in situ exsolved	BaZr_0.9_Y_0.1_O_2.95_	0.27	0.71	This work
BaCo_0.4_Fe_0.4_Zr_0.1_Y_0.1_O_3-__δ_	BaCe_0.7_Zr_0.1_Y_0.1_Yb_0.1_O_3-__δ_/1%wt. NiO	0.4	0.84	9
BaGd_0.8_La_0.2_Co_2_O_6-δ_	BaZr_0.7_Ce_0.2_Y_0.1_O_3-δ_	0.2	0.78	21
La_0.5_Ba_0.5_CoO_3-δ_	BaZr_0.9_Y_0.1_O_2.95_	0.33		16
